# Nabais Sa‐de Vries syndrome in a Chinese infant associated with a novel *SPOP* mutation: A clinical study and genetic report

**DOI:** 10.1002/mgg3.2075

**Published:** 2022-10-19

**Authors:** Wenjing Hu, Hongjun Fang, Yu Peng, Li Li, Shulei Liu, Hongmei Liao, Jingwen Tang, Jurong Yi, Qingqing Liu, Li Xu, Liwen Wu

**Affiliations:** ^1^ Department of Neurology Hunan Children's Hospital Changsha China; ^2^ Pediatrics Research Institute of Hunan Province Hunan Children's Hospital Changsha China; ^3^ Department of Radiology Hunan Children's Hospital Changsha China

**Keywords:** Nabais Sa‐de Vries syndrome, Novel mutation, NSDVS, Speckle‐type BTB/POZ protein, SPOP

## Abstract

**Background:**

Nabais Sa‐de Vries syndrome (NSDVS) is a newly identified neurodevelopmental disorder (NDD), characterized by mutations in the *SPOP* gene, which encodes the speckle‐type BTB/POZ protein. It is divided into two disease subtypes, according to patient facial features, which could be related to altered SPOP protein function. Few studies have documented this syndrome and little is known about its pathophysiology. Herein, we present an unexplained infant case of NDD, possibly the first Asian NSDVS case report.

**Methods:**

A 7‐month‐old boy presented with an enlarged head circumference, widened eye distance, and a protruding nose. Trio‐whole exome sequencing of the patient's family was performed, and a variant was identified by bioinformatics analysis and further verified by Sanger sequencing. This variant was then identified by molecular dynamics analysis. Finally, a plasmid was constructed in vitro to transfect the human 293 T cells. qPCR and western blotting (WB) experiments were subsequently performed. These analyses verified the variant's transcription and protein expression.

**Results:**

Trio‐whole exome sequencing was used to identify the *SPOP* mutation c.67 T > C (p.Cys23Arg). Crystal structure simulations suggest that this single‐residue substitution alters hydrogen bonding with nearby residues. Analysis via qPCR and WB experiments indicated decreased mutant mRNA and protein expression levels.

**Conclusion:**

Our findings suggest that genetic testing should be performed as soon as possible for children with NDD showing low phenotypic specificity. Prompt testing will provide more accurate diagnoses, which in turn offers evidence to assist in the formulation of rehabilitation training plans, and genetic counseling for patients' families.

## Graphical abstract

Nabais Sa‐de Vries syndrome (NSDVS) is a newly identified neurodevelopmental disorder (NDD). To date, only one study has been published, so little is known. Mutations in the SPOP gene, encoding the speckle‐type BTB/POZ protein, have been shown to cause NSDVS, but patients present with varied phenotypes. We present an unexplained infant case of NDD, possibly the first Asian NSDVS case report.

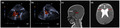

## 1 BACKGROUND

Protein homeostasis plays a crucial role in intracellular biological effects. The ubiquitin‐proteasome pathway (UPP) participates in intracellular protein homeostasis by regulating the degradation of most proteins (Tan et al., 2019). The speckle‐type BTB/POZ protein, encoded by the *SPOP* (OMIM#602650), promotes target protein degradation via UPP. This includes oncogene protein product degradation in several malignancies, such as prostate and endometrial cancer. Thus, somatic *SPOP* mutations seem to play a role in cancer occurrence and progression (Cotter & Rubin, 2022; Komander & Rape, 2012; Zhu et al., 2022). Furthermore, *SPOP* participates multiple organ development (Claiborn et al., 2010; Liu et al., 2009).

In 2020, Nabais Sá et al. reported that 7 children with developmental and intellectual disabilities carried sporadic *SPOP* mutations. In turn, this disorder was called Nabais Sa‐de Vries syndrome (NSDVS). Currently, this is the only study that reported the association between *SPOP* mutations and NDD (Nabais Sá et al., 2020). This disease can be further divided into subtypes based on patient facial features namely: NSDVS, type 1 (NSDVS1, OMIM#618828) and NSDVS, type 2 (NSDVS2, OMIM#618829). Patients affected by NSDVS1 display microcephaly, depressed nose bridge, and a small jaw. On the contrary, patients affected by NSDVS2 display and enlarged head circumference, broad forehead, low set ears, protruding nose bridge, and a bulbous nose. This variance could be caused by different manifestations of the mutant SPOP protein (Nabais Sá et al., 2020). However, due to the small number of reported cases, further studies are needed to clarify the correlation between disease phenotype and genotype.

Herein, we performed genetic diagnosis on an infant with NDD in a Chinese family. We identified a novel missense mutation in *SPOP*. Our findings further expand the phenotype and mutation spectrum of NSDVS caused by *SPOP* mutations.

## 2 MATERIALS AND METHODS

### 2.1 Subject

The case described here is from a nonconsanguineous family. The patient's parents provided informed consent. All studies were approved by the medical ethics committee of Hunan Children's Hospital.

### 2.2 Whole‐exome sequencing and Sanger sequencing

Trio‐whole exome sequencing (trio‐WES) was performed on DNA isolated from the patient and his parents. From 3 ml peripheral blood (using EDTA as the anticoagulant), leukocyte DNA was extracted using a kit according to the manufacturer's instructions (CoWin Biosciences Inc., Beijing, China). After library construction, sequencing was performed using an Illumina Noveseq 6000 high‐throughput sequencer (Illumina Inc., San Diego, CA, USA). The sequencing covered exons, with some intronic regions, implicated in more than 20,000 monogenic diseases. For suspicious variants, we checked for their presence in databases of the normal population, including dbSNP (www.ncbi.nlm.nih.gov/snp), ExAC (www.exac.broadinstitute.org/), and 1000 Genomes (www.1000genomes.org). We used GATK software to analyze single‐nucleotide, insertion, deletion, and other variants. After filtering out invalid variants, a hazard prediction analysis was carried out on the reliable variant spectrum using SIFT (www.sift.bii.a‐star.edu.sg); Polyphen‐2 (www.genetics.bwh.harvard.edu/pph2); and MutationTaster (www.mutationtaster.org) software. Finally, variant pathogenicities were rated by the American College of Medical Genetic (ACMG) guidelines (Richards et al., 2015). For suspected variants, primers were designed using the Ensemble database for Sanger sequencing using an ABI 3500XL analyzer (Thermo Fisher Scientific, Inc., Waltham, MA, USA).

### 2.3 Molecular dynamics

We downloaded the SPOP crystal structure from PDB (3HQI) (https://www.ncbi.nlm.nih.gov/nuccore/NM_001370730.1/). A three‐dimensional, full‐length, tertiary structure was constructed using Modeler 10.1 software (https://salilab.org/modeller/). The mutant structure was constructed using PyMOL 2.5 software (https://pymol.org/2/). Wild‐type and mutant protein structures were analyzed using MD for 10 ns, under constant pressure and constant temperature, using the GROMACS 5.1.4 (http://www.gromacs.org/) software. Structural parameters were obtained after optimization using the AMBER FF99SB force field (Abraham et al., 2015). Finally, Chimera 1.15 software (http://www.cgl.ucsf.edu/chimera/) was used to analyze the interaction after local optimization.

### 2.4 Cell culture and plasmid construction

Human 293 T cells were obtained from the Kunming Cell Bank of Chinese Academy of Sciences (Kunming, China) were cultured in high‐glucose DMEM (Gibco Inc., Waltham, MA, USA) containing 10% fetal bovine serum in 5% CO_2_, at 37°C, in six‐well plates, to 60% confluence. Lipofectamine 2000 (Thermo Fisher Scientific Inc., Carlsbad, CA, USA) was used for plasmid transfection. We used the pECMV‐3 × FLAG‐N vector to construct a SPOP‐WT expression plasmid by PCR using a Phanta® Max Super‐Fidelity DNA Polymerase (Vazyme #P505) kit. Primers were: 5′‐ATGACAAGCCTTGGTACCGAGCTCGGATCCATGCAGGGTTCCAAGTCCACCTC‐3′; 5′‐CGGGTTTAAACGGGCCCTCTAGGACTCGAGTTAGGATTGCTTTCAGGCGTTTGGGGGGGGG‐3′. We mutagenized the resulting plasmid to produce SPOP‐MUT using a Mut Express MultiS Fast Mutagenesis Kit V2 (Vazyme #C215, Nanjing, China), using primers: 5′‐AGAGTTGGCGCTACACAGATCAAGGTAGTGTGAAA‐3′; 5′‐TGTGTAGCGCCAACTCTCAGCTACGGGGCCAC‐3′. All constructs were verified by sequencing.

### 2.5 RT‐PCR

At 48 h following transfection, cells were lysed with TRIzol reagent (Thermo Fisher Scientific, Inc., Waltham, MA, USA). RNA was extracted and analyzed by qPCR (Applied Biosystems 7500 Fast Real‐Time PCR System, Thermo Fisher Scientific, Inc., Waltham, MA, USA) using TIANScript II M‐MLV reverse transcriptase (Tiangen, Inc., Beijing, China; #ER107) and the FastFire qPCR PreMix (Tiangen, Inc., Beijing, China; #FP208). Reverse transcription was performed at 42°C for 60 min; the reaction was stopped by heating to 70°C for 10 min. PCR conditions were 95°C for 10 min; 40 cycles of 95°C 30 s and 60°C 30 s; and 72°C 30s. The last 95°C 15 s. CT values were calculated by the relative quantitative method 2^−ΔΔCt^, using GAPDH expression for normalization.

### 2.6 Western blotting

After cells transfected with SPOP‐WT and SPOP‐MUT were lysed using RIPA lysis buffer (Beyotime, Inc., Shanghai, China), protein expression was quantified by western blotting and analysis using ImageJ software. Primary antibodies were mouse anti‐human FLAG (#8146) and anti‐GAPDH (#97166, both from Cell Signaling Technology, Inc., Danvers, MA, USA), diluted 1:1000. The secondary antibody was anti‐mouse IgG, HRP‐linked (#7076, also from Cell Signaling Technology), diluted 1:5000.

## 3 RESULTS

### 3.1 Case presentation

**FIGURE 1:**
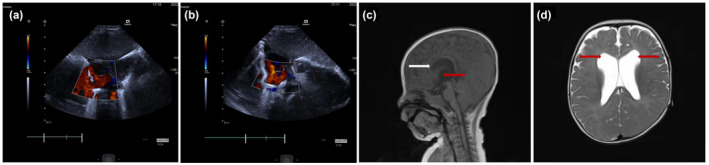
Clinical features. (a,b) Color Doppler echocardiography showing lac of closure of the foramen ovale; the procedure was interrupted at the foramen ovale of the atrial septum, with multiple strands of left to right septal blood were seen in the middle of the atrial septum, the widest of about 3 mm (10 days old). (c) Cranial MRI at 7 months old showing enlargement of the third ventricle (red arrow) and thinning of the corpus callosum (white arrow). (d) Bilateral ventricular enlargement (red arrow)

The patient was a 7‐month‐old boy to nonconsanguineous parents of Chinese Han ancestry. He was born at 38^+2^ weeks by cesarean section to a gravida 2 para 2 woman. His birth weight was 3300 g. The baby's Apgar scores were unknown. At 10 days after birth, he required phototherapy for neonatal jaundice. Moreover, otoacoustic emissions indicated abnormal binaural hearing. An echocardiography revealed a patent foramen ovale and tricuspid regurgitation (Figure 1a,b). Upon admission at 7 months of age, the patient's height was 66.5 cm (−1 standard deviation, *SD*), weight was 7.8 kg (−1 *SD*), and head circumference was 46.5 cm (+1.5 *SD*). His face was characterized by increased interocular distance, low set ears, protruding nose, sharp chin, and an inverted triangular face. His limb tension was high, about level 1+. Furthermore, his movement and developmental milestones were delayed. When placed in a supine position, his posture was found to be asymmetric, and the patient could not turn over. There was also no displacement and straightening posture when placed in a prone position. His head control was poor, as there were clear signs of head lag. The patient could stand for a short time (Table 1). The Gesell adaptive behavior development quotient (DQ) score was 47, grand motor score DQ was 23, fine motor score DQ was 52, language score DQ was 45, and personal social behavioral score DQ was 41. Brain magnetic resonance imaging showed enlargement of the bilateral lateral ventricles and the third ventricle, with the frontal horn of the bilateral lateral ventricles and the anterior part of the body obviously deformed. The corpus callosum was slightly thin (Figure 1c,d). Both parents were healthy and had no knowledge of any family genetic history of NDD.

**TABLE 1:** Clinical and molecular data of individuals with NDD caused by reported germline *genetic* mutation of *SPOP* gene

Findings		Nabais Sá et al. (2020)	Nabais Sá et al. (2020)	Nabais Sá et al. (2020)	Nabais Sá et al. (2020)	Nabais Sá et al. (2020)	Nabais Sá et al. (2020)	Nabais Sá et al. (2020)
Proband ID	Present study	1	2	3	4	5	6	7
Sex	Male	Female	Male	Female	Male	Male	Female	Female
Current age	7 months	4 year 7 month	10 year	10 month	17 year 11 month	17 year 9 month	20 year	15 year
Facial features								
Head	−	Brachycephaly	Narrow forehead	Frontal bossing	−	Frontal bossing	Frontal bossing	Frontal bossing
Eyebrow	−	Arched	Arched	−	−	Sparse	Sparse	−
Eye	Hypertelorism	Blepharophimosis, enophthalmos	Long eyelashes, blepharophimosis, epicanthus	Hypertelorism, enophthalmos	Hypertelorism	Hypertelorism, Eyelid fissure length	Hypertelorism	Asymmetric palpebral fissure
Nose	Protruding nasal bridge, leaning nostrils forward, spherical nose tip	Protruding nasal bridge, bulbous nasal tip and hypoplasia of nasal wing	Low nasal bridge, spherical nose tip, short nose	Low nasal bridge, leaning nostrils forward	−	Protruding nasal bridge, spherical nose tip	Nasal bridge is wide and protruding, spherical nose tip	Protruding nasal bridge
Mouth and chin	Flat philtrum	Flat philtrum, pointed chin	Flat philtrum, pointed chin	Broad gums, micrognathism	Gothic arch, pointed chin	Pointed chin	Bilateral cleft lip and palate	Thin lip
Growth development								
Height (centile range)	66.5 cm (−1 *SD*)	104.5 cm (~50th)	77 cm (10th–25th)	65.1 cm (−2.5 *SD*)	151.8 cm (−3.1 *SD*)	178.5 cm (50th‐75th)	172 cm (90th–97th)	158.8 cm (25th–50th)
Weight (centile range)	7.8 kg (−1 *SD*)	15.3 kg (10th–25th)	8.8 kg (−2.3 *SD*)	5.6 kg (−4 *SD*)	49.7 kg (3rd)	73 kg (50th–75th)	89 kg (97th)	90.7 kg (+2.5 *SD*)
Head circumference (centile range)	46.5 cm (+1.5 *SD*)	44 cm (‐4 *SD*)	40.5 cm (−5 *SD*)	49 cm (+3.5 *SD*)	NA (25th)	56.4 cm (75th–90th)	57 cm (+2.5 *SD*)	56 cm at 5 year (+4 *SD*)
Mental development								
Motor retardation	+	+	+	+	+	+	+	+
Language development delay	+	+	+	+	+	+	+	−
Intellectual disability	Moderate		Serious		Moderate	Moderate	Moderate	Mild
Nervous system characteristics								
Abnormal brain structure	Enlargement of bilateral ventricles and third ventricle, thin corpus callosum	Simplified gyri	NA	Ventricular enlargement	Ventricular enlargement, thin corpus callosum	−	NA	−
Behavior characteristics	Unconcerned	Social phobia	Autoagression	NA	ADHD, agitation	Autism	Irritable	Anxiety
Seizures	−	−	NA	−	+	+	NA	−
Other phenotypes								
Ophthalmologic	−	−	Bilateral optic nerve hypoplasia	Small optic disc	Abnormality of refraction	−	Strabismus	Abnormality of refraction
Hearing	Bilateral hearing loss	Sensorineural hearing loss	Bilateral hearing loss	−	−	−	−	−
Cardiovascular	Patent foramen ovale, tricuspid regurgitation	−	−	Ventricular septal defect, pulmonary valve stenosis and supravalvular pulmonary valve stenosis, mild right ventricular outflow tract obstruction	Hypoplastic left heart syndrome with left ventricular outflow tract obstruction	NA	ASD	Patent ductus arteriosus, ventricular septal defect
Urogenital	−	−	Bilateral vesicoureteral reflux	−	Hypogonadism	+	NA	Left polycystic kidney
Nucleotide variation	c.67 T > C	c.362G > A	c.430G > A	c.395G > T	c.73A > G	c.412C > T	c.412C > T	c.248A > G
Amino acid change	p.Cys23Arg	p.Arg121Gln	p.Asp144Asn	p.Gly132Val	p.Thr25Ala	p.Arg138Cys	p.Arg138Cys	p.Tyr83Cys
Inheritance	de novo	de novo	de novo	de novo	de novo	de novo	de novo	de novo
Subtypes	2	1	1	2	2	2	2	2

### 3.2 Genetic testing

**FIGURE 2:**
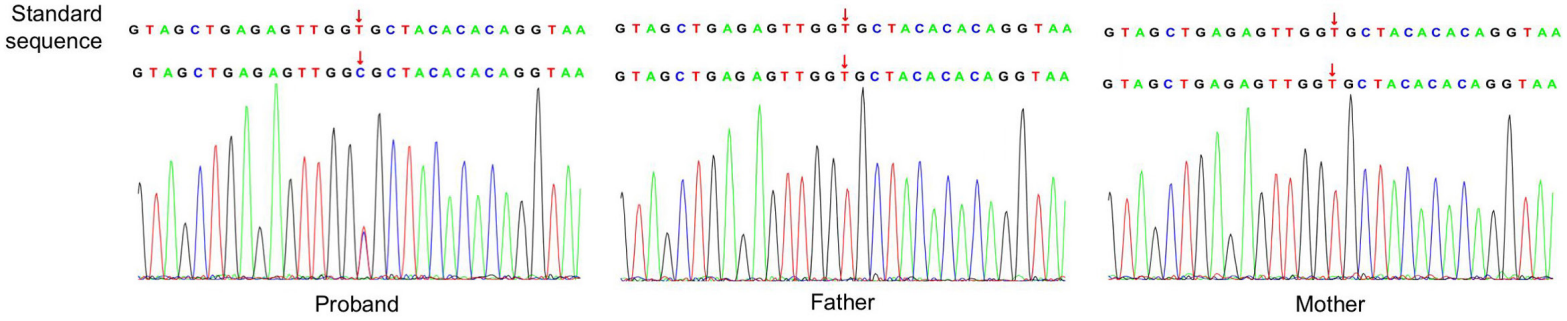
Identification of the SPOP (NM_001370730.1) heterozygous missense mutation c.67 T > C (p.Cys23Arg). Both of the patient's parents are homozygous wild type

Using WES, 50.9 million clean reads were obtained, the average sequencing depth was 112.43X, and the average coverage of target regions larger than 20X was 99.10%. Finally, a heterozygous novel missense mutation in exon 3 of *SPOP*, NM_001370730.1: c.67 T > C (p.Cys23Arg). Both parents were wild type for both alleles, so this mutation is likely to have been sporadic. Sanger sequencing confirmed the existence of this mutation (Figure 2), which was not present in the dbSNP, ExAC, or 1000 Genomes databases. We used online software (SIFT, Polyphen‐2 and MutationTaster) to predict the pathogenicity of this mutation. According to the rating rules from ACMG guidelines, the Cys23Arg was defined as pathogenic with characteristics PS2 + PM1 + PM2 + PP2 + PP3.

### 3.3 Cys23Arg alters SPOP protein structure

**FIGURE 3:**
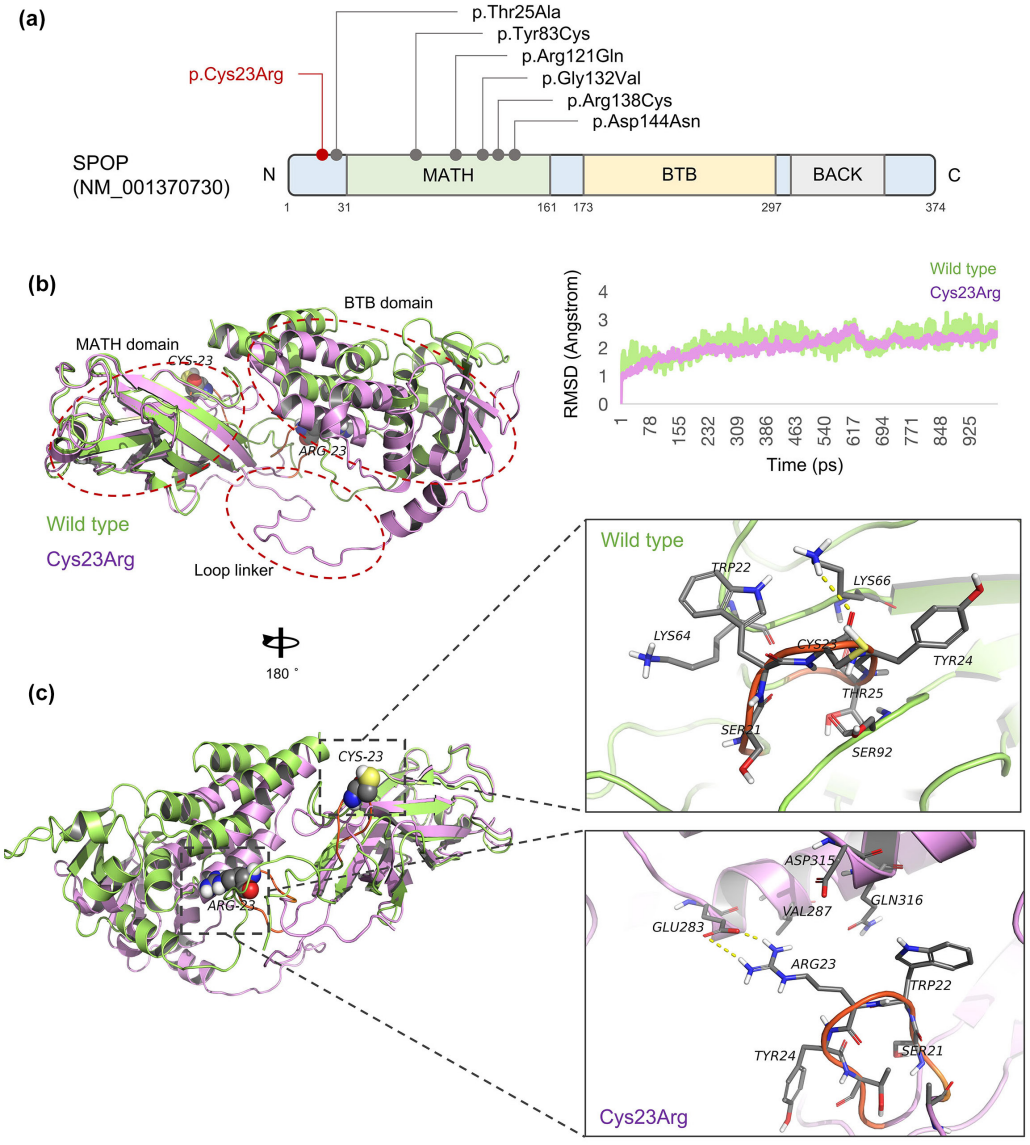
The SPOP Cys23Arg substitution alters protein structural predictions. (a) SPOP domain structure showing positions of mutations. The Cys23Arg substitution (red font) is in the N‐terminal loop adjacent to the MATH domain. (b) Wild‐type SPOP (green) Cys23 interacts with Lys66 in the MATH domain. The Cys23Arg substitution (purple) flips the loop linker closer to the BTB domain. (c) Mutant (purple) Cys23Arg destroys the hydrogen bond with Lys66 in the MATH domain and forms a new hydrogen bond with Glu283 in the BTB domain, changing local structure

SPOP includes N‐terminal MATH, intermediate BTB, and C‐terminal BACK domains (10). Cys23 is a loop region near the MATH domain; its main chain carbonyl forms a hydrogen bond with Lys66 of the MATH domain. MD simulations suggested that with the Cys23Arg substitution, the loop flips 180 degrees and forms a hydrogen bond with Glu283 in the BTB domain. Due to this structural change, both expression and function are likely to be affected (Figure 3).

### 3.4 The c.67 T > C/p.Cys23Arg mutation alters both mRNA and protein expression levels

**FIGURE 4:**
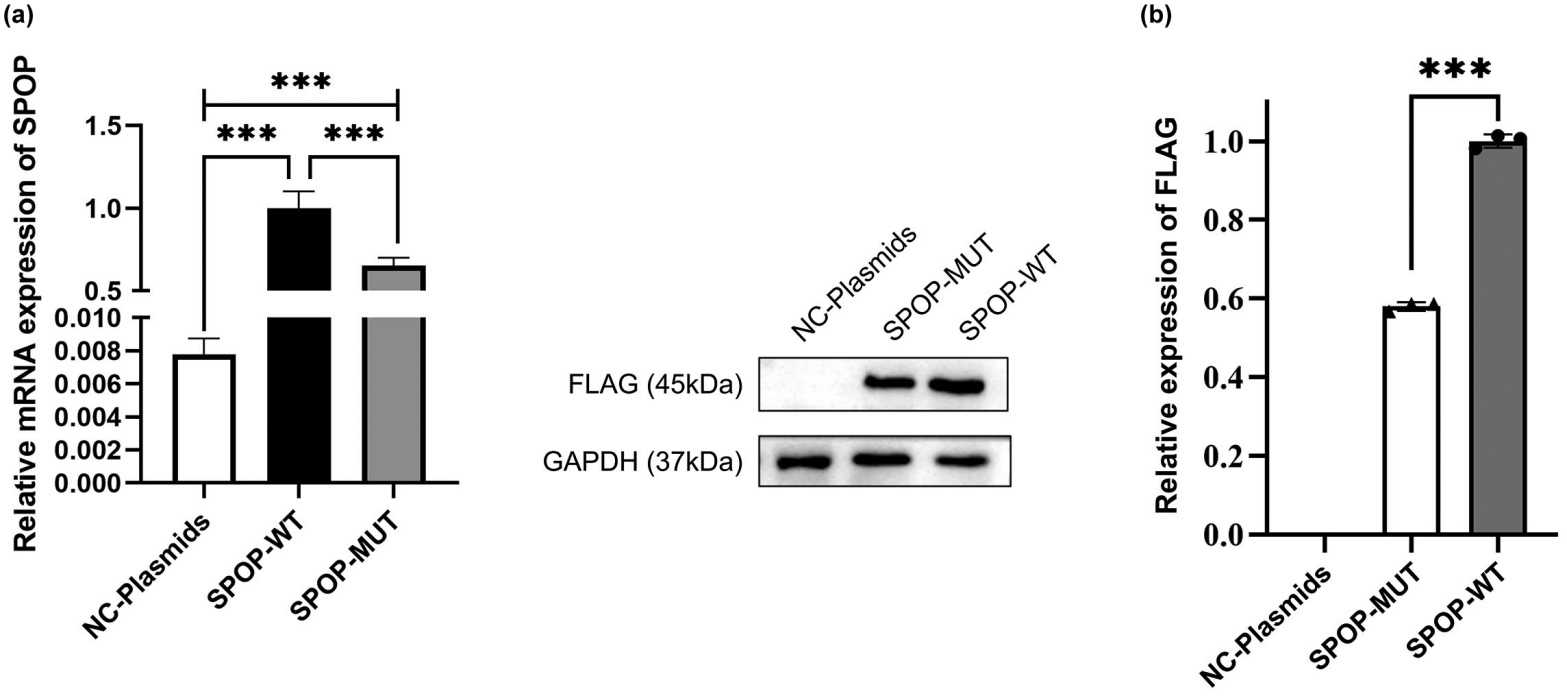
The SPOP Cys23Arg substitution decreases both mRNA and protein expression levels in transfected cells. (a) Expression of SPOP‐MUT mRNA was decreased relative to SPOP‐WT (*p* = .006). (b) Western blotting showing decreased protein expression from the SPOP‐MUT plasmid relative to that from the SPOP‐WT plasmid (*p* < .001)

Cells transfected with the SPOP‐WT and SPOP‐MUT group showed significant expression but mRNA expression from the SPOP‐MUT plasmid was significantly lower than that from the SPOP‐WT plasmid, with a decrease of about 35% (*p* = .006) (Figure 4a). Both plasmids produced SPOP protein, but the expression level from SPOP‐MUT was 42% lower than that from SPOP‐WT (*p* < .001) (Figure 4b).

## 4 DISCUSSION

To our knowledge, this is the first report of NSDVS in Asian populations. Our patient had typical NDD symptoms, including psychomotor retardation, delayed motor milestones, intellectual disability, wide eye distance, protruding nose, and sharp chin (Nabais Sá et al., 2020). Our patient also had bilateral hearing impairment, congenital heart disease, and brain structural abnormalities. We identified a heterozygous novel variant of *SPOP*. The mutant Cys23Arg is located in the N‐terminal loop region of SPOP protein. Our MD simulations suggested local structural changes in the SPOP protein. In cultured, transfected cells, the mutation led to decreased protein expression.

To date, only seven cases of NDD caused by *SPOP* mutations have been reported; two disease subtypes can be distinguished by facial features (Nabais Sá et al., 2020). For example, two cases with microcephaly, sunken bridge of the nose, and small jaw were diagnosed as NSDVS1; The other five cases, presenting with increased head circumference, low ear position, protruding nose bridge, and bulbous nose, were diagnosed as NSDVS2. Published studies have suggested that different NSDVS subtypes may be caused by functional differences among *SPOP* mutations. A total of six *SPOP* mutations have been identified in seven reported NSDVS patients; five alter the SPOP MATH domain, with one altering the N‐terminal loop (Figure 2a). In NSDVS1, *SPOP* mutations increase degradation of bromodomain and extraterminal (BET) domain proteins, substrates of SPOP. In dominant NSDVS2, BET proteins are stabilized (Nabais Sá et al., 2020). At present, although it is not clear how the functional changes of SPOP protein lead to differences in facial phenotypes between subtypes, additional NSDVS reports will aid in understanding subtype classification. In addition to low ear position, protruding nasal bridge and sharp chin, our patient presented with ventricular enlargement and congenital heart disease. Since our studies in transfected cells showed decreased mutant of protein expression, we diagnosed our patient with NSDVS2.

The speckled‐type BTB/POZ protein encoded by SPOP has a total of 374 resides. Its main components are an N‐terminal MATH, a middle BTB, and an C‐terminal BACK domain, performing different biological functions (Hernández‐Muñoz et al., 2005). Six of the reported *SPOP* mutations are in the MATH domain (5/6), which recruits substrate proteins through SPOP binding partners that specifically recognize substrates (Janouskova et al., 2017; Mani, 2014). It is worth noting that prostate cancer and endometrial cancer caused by somatic *SPOP* variants tend to have alterations in the MATH domain. The dominant‐negative effects of *SPOP* mutant proteins on different substrate proteins may correspond to the occurrence and progression of endometrial and prostate cancers (Baca et al., 2013; Barbieri et al., 2012; Janouskova et al., 2017; Le Gallo et al., 2012). Therefore, our patient's missense mutation in the MATH domain leads to altered binding with substrate, which further indicates that this domain plays a key functional role. Although Cys23Arg, the novel *SPOP* substitution identified in our patient, is not in the MATH domain, it likely affects the structural stability of both the MATH and BTB domains. The BTB domain binds primarily to the ubiquitin ligase cullin 3 and promotes the homodimerization of SPOP (Mani, 2014). In addition, one reported substitution (Thr25Ala) is close to Cys23Arg; patients with Thr25Ala have phenotypes similar to those of our patient, but there was neither macrocephaly nor striking nasal morphologies (Nabais Sá et al., 2020). This suggests that loop mutations lead to mild clinical features, especially facial ones. This increases the difficulty of clinical diagnosis of craniofacial malformations.

The mechanism underlying *SPOP* regulation of nervous‐system development is still unclear; however, it may involve the inhibition of the transcription factor Ci (Gli) protein in the Hedgehog (HH) signaling pathway that affects neuronal patterning (Briscoe & Thérond, 2013; Cai & Liu, 2017). There is also evidence that neuroepithelial cells in mice lacking the BET protein Brd2 are damaged in the process of neuronal differentiation and cell‐cycle exit. Therefore, if a *SPOP* mutation reduces BET expression due to function acquisition, neuronal differentiation will be affected, causing microcephaly. Conversely, reduced SPOP function should increase BET expression, which may alter head development (Li et al., 2016; Tsume et al., 2012). With increasing in‐depth studies, researchers in the future will be better able to identify key mechanisms and to design more effective interventions, which is of great significance for improving the treatment of NDD.

In conclusion, we found an Chinese infant presenting with NSDVS2 caused by Cys23Arg, a novel *SPOP* mutation. Compared with NSDVS1, NSDVS2 tends to cause macrocephaly, and increased eye‐to‐eye width, protruding nasal bridge, bulbous nose, and sharp chin. The increased head circumference in patients with NSDVS2 may be a dominant‐negative effect caused by *SPOP* mutation. We found that the Cys23Arg substitution is likely to produce local structural changes of the protein. Our findings enrich the mutation spectrum of *SPOP*. However, our study still has shortcomings, such as the lack of BET protein degradation experiments and long‐term clinical follow‐up. We will continue to track disease progression in the patient, and design more targeted neurological rehabilitation training to improve his quality of life.

## AUTHOR CONTRIBUTIONS

WH, HF, and LW initiated the study, obtained clinical data and analyzed it, and wrote the manuscript and critically reviewed it. WH, LL, SL, HL, and LX obtained clinical data and analyzed it. YP, HL, and JT performed and analyzed the experimental studies. YP, JY, and QL interpreted the exome sequencing data. All authors critically reviewed the manuscript and approved the submitted version.

## FUNDING INFORMATION

This work was supported by the National Natural Science Foundation of China (Grant No. 82171453), the National Natural Science Foundation of Hunan province (Grant No. 2022JJ70087), Clinical Medical Technology Innovation Guidance Project of Hunan Province (Grant No. 2021SK50512).

## CONFLICT OF INTEREST

The authors declare that the research was conducted in the absence of any commercial or financial relationships that could be construed as a potential conflict of interest.

## ETHICS APPROVAL AND CONSENT TO PARTICIPATE

The study was approved by the ethics committee of the Hunan Children's Hospital. Written informed consent was provided by the participant.

## Supporting information


**Supinfo S1** Original blot gel picture

## Data Availability

The data that support the findings of this study are available from the corresponding author upon reasonable request.
