# Supplementary figures and images for: Nabais Sa‐de Vries syndrome in a Chinese infant associated with a novel SPOP mutation: A clinical study and genetic report
Source: Mol Genet Genomic Med. 2022 Oct 19;10(12):e2075. doi: 10.1002/mgg3.2075 (PMC9747555; doi:10.1002/mgg3.2075)

**Supplementary materials**

Original blot gel picture

**
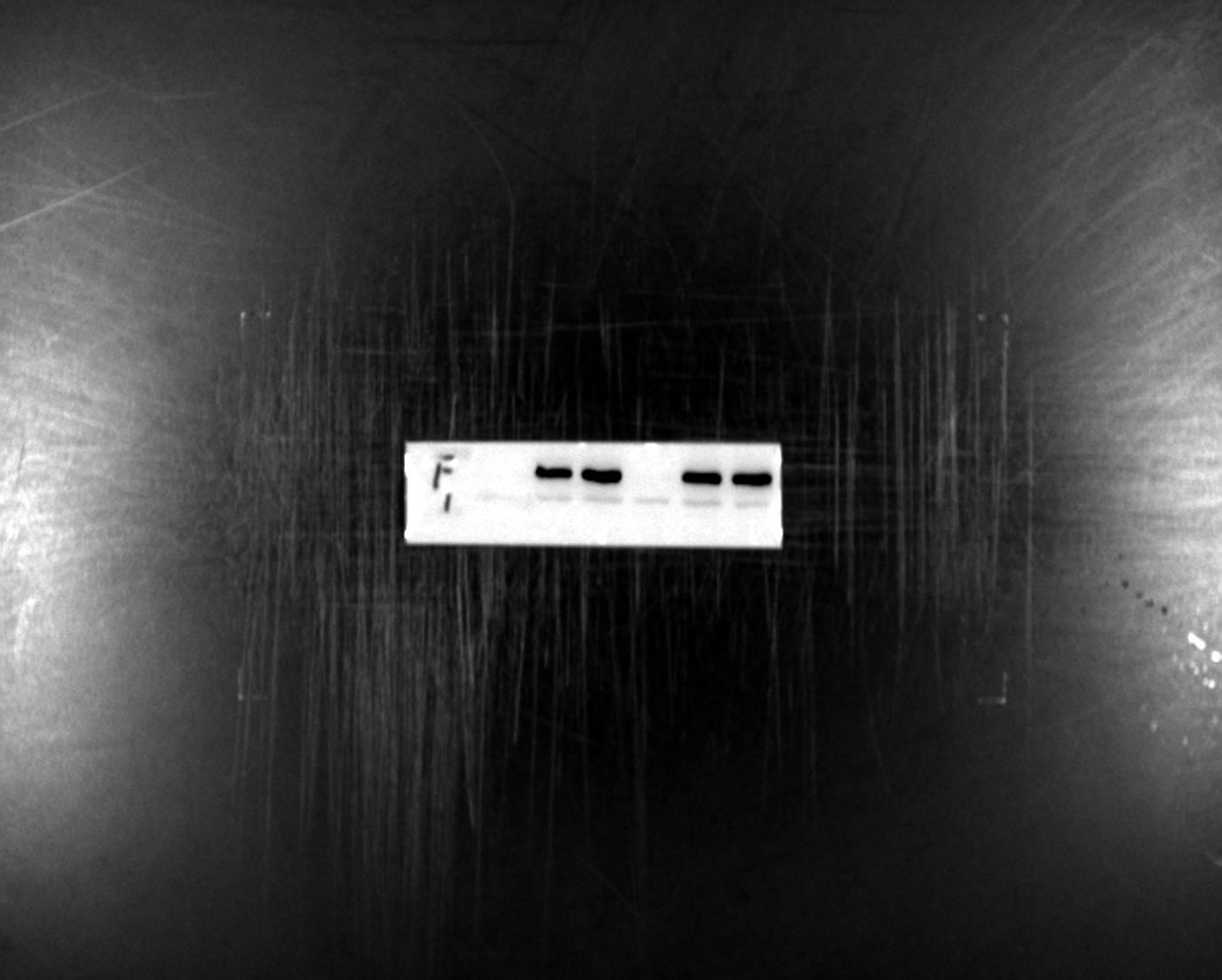

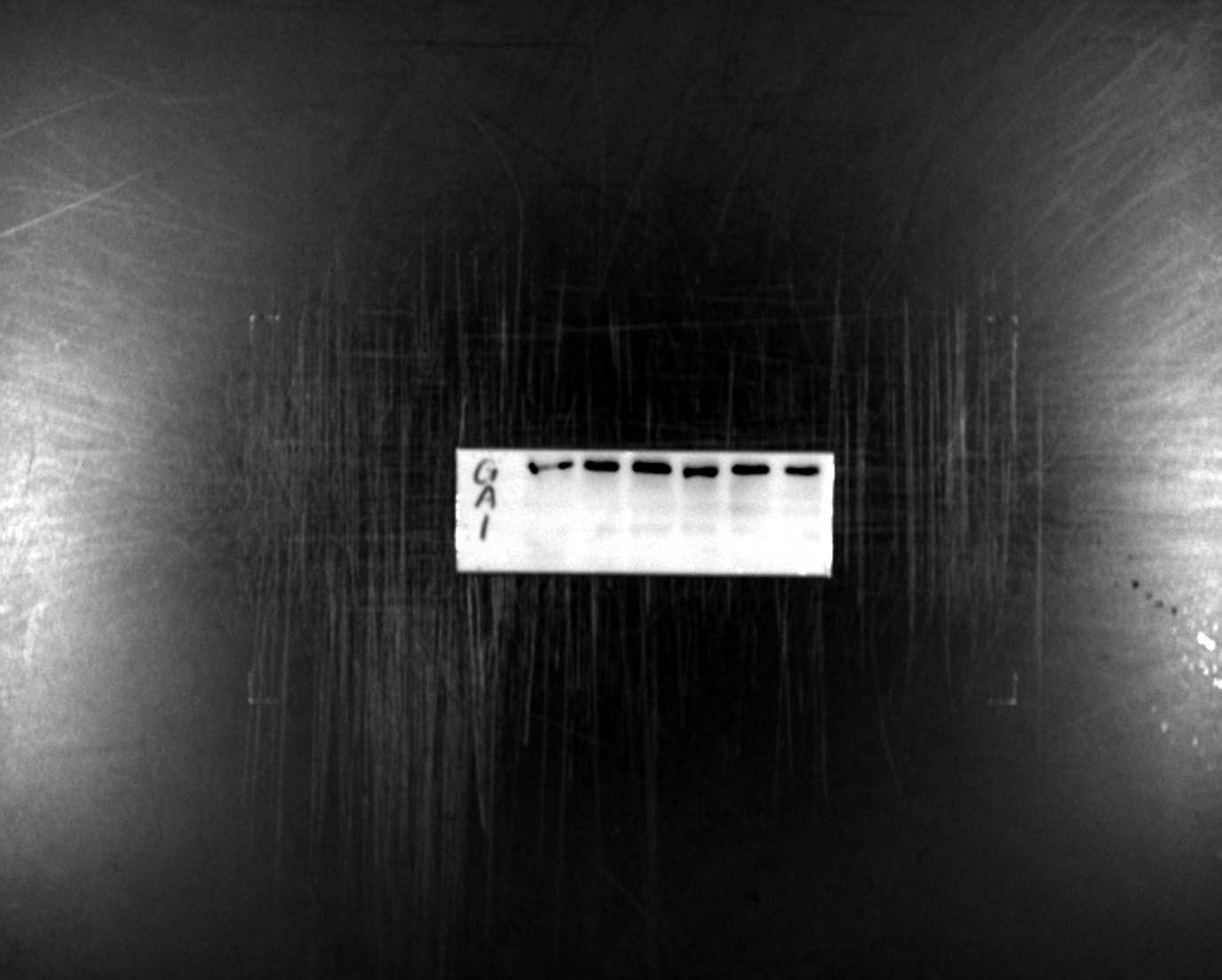
**

**FLAG GADPH**

Supplement: Supplementary file 1 — Supinfo S1 Original blot gel picture [file MGG3-10-e2075-s001.docx]
